# Clinical study evaluating the gastroprotective effect of carvedilol in patients with ischemic heart disease on aspirin therapy

**DOI:** 10.1007/s10787-025-01961-1

**Published:** 2025-09-30

**Authors:** Sarah M. Elkablawy, Aliaa E. Shaban, Tarek M. Mostafa

**Affiliations:** 1 Clinical Pharmacy Department, Faculty of Pharmacy, Tanta University, Tanta, 31527 Egypt; 2 Cardiovascular Department, Faculty of Medicine, Tanta University, Tanta, Egypt

**Keywords:** Carvedilol, Gastroprotective, 4-HNE, GAS-17, PGE2, SAG-7

## Abstract

**Background and purpose:**

Despite its therapeutic benefits in ischemic heart disease (IHD) patients, using aspirin represents a significant risk of gastric ulcers. Therefore, this study aimed to inspect the gastroprotective potential of carvedilol in IHD patients undergoing aspirin treatment.

**Patients and methods:**

In this randomized, controlled parallel trial, 66 patients with IHD on aspirin therapy were assigned to group 1 (control, *n* = 33), received aspirin 150 mg plus captopril 12.5 mg twice daily and standard IHD medications, and group 2 (carvedilol group, *n* = 33), received aspirin 150 mg plus carvedilol 12.5 mg twice daily and standard IHD medications for three months. All patients were subjected to assessments for demographic data, anthropometric measurements, and biochemical measurements of the serum levels of malondialdehyde (MDA), 4-hydroxynonenal (4-HNE), prostaglandin E2 (PGE2), and gastrin-17 (GAS-17). The researchers also evaluated the Structured Assessment of Gastrointestinal Symptoms (SAGIS) questionnaire and the Seattle Angina Questionnaire (SAQ-7) to assess changes in the quality of life (QOL).

**Results:**

Three months post-treatment and relative to the control group, the carvedilol group exhibited significantly reduced serum levels of MDA (*P*2 = 0.003), 4-HNE (*P*2 < 0.001), and GAS-17 (*P*2 = 0.015), which was associated with significantly higher serum levels of PGE2 (*P*2 < 0.001). Additionally, the carvedilol group showed a significantly higher SAQ-7 score (*P*2 = 0.033) and a significantly lower SAGIS questionnaire score (*P*2 = 0.04) than the control group.

**Conclusion:**

Carvedilol could represent a potential gastroprotective agent for patients with IHD on aspirin therapy secondary to its efficacy and safety.

*Clinicaltrial.gov ID*: NCT05553717.

## Introduction

Gastric ulcers are a prevalent gastrointestinal tract (GIT) condition, affecting around 4 million people in the world annually, with complications occurring roughly 10–20% (Zibima et al. 2020). These ulcers result from an imbalance between mucosal defense mechanisms—such as prostaglandin production, mucosal blood flow, and nitric oxide release—and harmful factors such as gastric acid, pepsin, and Helicobacter pylori infection. Clinical manifestations include mucosal erosion, bleeding, and perforation (Shell 2021). The primary causes of peptic ulcers are infections by *H. pylori*, frequent usage of nonsteroidal anti-inflammatory drugs (NSAIDs), or both (Kavitt et al. 2019). Aspirin (acetylsalicylic acid) is routinely used for its cardioprotective effects in ischemic heart disease (IHD) patients, particularly in the prevention of thrombotic cardiovascular events such as myocardial infarction (Cadavid 2017). Chronic use of low dose aspirin significantly linked to gastrointestinal toxicity, particularly gastric ulceration (Chitapanarux et al. 2019; Sung et al. 2010; Kamada et al. 2021; Lanas 2011; Sorensen et al. 2000; Yeomans et al. 2008) The mechanism underlying aspirin-induced gastric ulcers involves cyclooxygenase (COX) inhibition, which suppresses prostaglandin synthesis. This reduction in PGs triggers multiple detrimental effects: diminished mucus and bicarbonate production, impaired mucosal perfusion, and disrupted platelet aggregation. Additionally, microvascular structural changes occur, promoting epithelial injury. These alterations are compounded by enhanced leukocyte adhesion, upregulation of pro-inflammatory cytokines, excessive reactive oxygen species (ROS) generation, and depletion of antioxidant defense systems (Abd El-Ghffar et al. 2018). Enteric-coated aspirin is associated with lower gastrointestinal toxicity; however, it exhibits a slower absorption rate compared to uncoated formulations. Moreover, the enteric coating may lead to reduced drug bioavailability (Angiolillo and Capodanno 2021) . Carvedilol exerts its cardiovascular effects through combined β- and α1-adrenoceptor inhibition, making it an effective treatment option for hypertension, cardiac insufficiency, and ischemic chest pain. Carvedilol exhibits metal-chelating properties and demonstrates mitochondrial protection against oxidative damage (Toyoda et al. 2020). Furthermore, carvedilol exhibits antioxidant and anti-inflammatory properties against renal, hepatic, and cardiac toxicity. Moreover, it is suggested that carvedilol may also counteract aspirin-associated gastrointestinal damage, including gastric ulceration (Osman et al. 2017). While research in this specific area remains limited, preliminary animal studies indicate promising results. A recent investigation reported that carvedilol administration improved gastric mucosal histopathology in cold stress-induced ulcer models, supporting its gastroprotective potential (Ahmed et al. 2020). These findings underscore the necessity for additional research to assess carvedilol's potential as a gastroprotective agent in aspirin-treated patients. In this context, this study aims to inspect the gastroprotective effect of carvedilol in ischemic heart disease (IHD) patients undergoing aspirin treatment.

## Patients and methods

### Study design and ethical approval

This research was a randomized, controlled parallel trial carried out at the Cardiovascular Diseases Department, Tanta University Hospital, from October 2022 to November 2023. The study was registered on ClinicalTrials.gov (ID: NCT05553717). The study protocol received formal approval from two independent ethics committees: the Research Ethics Committee of Tanta University (approval code: 35741/9/22) and the Research Ethics Committee of the Faculty of Pharmacy, Tanta University (approval code: TP/RE/1/25M-002). Prior to enrollment, all participants underwent a comprehensive study briefing and provided written informed consent. This investigation was conducted in full adherence to the ethical principles outlined in the 1975 Declaration of Helsinki.

### Study protocol and randomization

Patients with IHD receiving aspirin treatment were assessed for eligibility and subjected to a thorough physical examination. The selected patients were enrolled and randomly assigned in a 1:1 ratio using a computer-generated code, following the Consolidated Standards of Reporting Trials (CONSORT) guidelines. They were allocated to receive either aspirin 150 mg tablets plus captopril 12.5 mg tablets twice daily for three months (control group; *n* = 33) or aspirin 150 mg tablets plus carvedilol 12.5 mg tablets twice daily for three months (carvedilol group; *n* = 33). All patients in the two groups were allowed to take their standard medications. The inclusion criteria were patients with IHD on low doses of aspirin (150 mg) treatment for ≥ 3 months, ages 25–60 years old, both sexes, and patients with hypertension. The exclusion criteria included patients with a history of gastrointestinal disorders, H. pylori infection, gastroduodenal surgery, major depressive disorder, or other psychiatric conditions. Additional exclusion criteria consisted of recent use (within two weeks) of acid-suppressive medications (proton pump inhibitors, histamine-2 receptor antagonists), misoprostol, or sucralfate; known hypersensitivity or intolerance to aspirin and NSAIDs; significant hepatic impairment (Child–Pugh class B or C); renal impairment (serum creatinine > 1.5 mg/dL); or current pregnancy or lactation.

### Methods

#### Demography, physical examination and anthropometeric data

At baseline, all participants were submitted to demography, physical examination, medical history review (concomitant use of steroids or concurrent dual antiplatelet/anti-coagulant therapy). Additionally, weight and height were quantified, and body mass index (BMI) was computed utilizing the formula: (BMI) = [Weight (kg) ÷ Height^2^ (m)]. Echocardiography (ECG) and measurements of blood pressure were also done.

#### Blood sample collection and biochemical measurements

Blood samples (6 mL each) were collected from the antecubital vein of all participants at baseline and three months after treatment, between 9:00 AM and 10:00 AM. The collected blood underwent 10-min centrifugation at 3000 rpm, then the serum was kept at −80 °C until analysis of the biological parameters. Double-antibody sandwich enzyme-linked immune-sorbent assay (ELISA) kits were used for the assessment of malondialdehyde "MDA" (Solarbio, beijing, China, Catalogue No:BC0020), 4-hydroxynonenal "4-HNE" (SunRed, Shanghai, China, Catalogue No:201-12-1979), prostaglandin E2 "PGE2" (SunRed, Shanghai, China, Catalogue No:201-12-1010) and gastrin-17 "GAS-17" (SunRed, Shanghai, China, Catalogue No:201–12-2036).

#### Clinical assessment

The assessment of clinical outcome was done using the seattle angina questionnaire (SAQ-7) questionnaire to evaluate the change in the quality of life (QOL) and functioning status in IHD patients and the structured assessment of gastrointestinal symptoms (SAGIS) questionnaire to evaluate the change in the severity of gastrointestinal symptoms.

#### Evaluation of medication safety and patients' adherence

During the study duration, the medications were supplied monthly, and adherence was monitored by counting the pills and by the medication refilling rate. Participants received weekly phone follow-ups and attended monthly clinic visits to track compliance and document any drug-related adverse effects using adverse effects reporting form.

### Primary and secondary outcomes

The primary outcome measured the improvement in the quality of life (QOL) and gastrointestinal symptoms. The Secondary outcome included variations in biomarker levels.

### Sample size calculation

Using G*Power software (version 3.1.0; Heinrich Heine University Düsseldorf, Germany) to estimate the minimum sample size required. Based on a two-tailed independent t-test to detect a large effect size (Cohen’s *d *= 0.92), with an alpha level (α) of 0.05 and statistical power (1 − *β*) of 0.90, the required sample size was determined to be 60 participants (30 per group). To account for an expected dropout rate of around 10%, the final sample size was augmented to 66 participants (33 per group). This calculation ensured adequate power to detect meaningful differences between the study groups.

### Statistical analysis

The statistical analysis was carried out with SPSS statistical package version 27.0 (December 2020) IBM corporation software group, USA. The data were tested for normality using Kolmogorov–Smirnov test or Shapiro Wilk test. Parametric data were analyzed by paired student *t*-test and unpaired t-test to compare the mean values within the same group and between the two groups before and after treatment. Non- Parametric data were analyzed by Mann–Whitney U test to compare the mean values within the same group and between groups before and after treatment. Categorical variables were evaluated with the Chi-square test, while Fisher’s exact test was applied for adverse event analysis. Pearson or Spearman correlation which appropriate were used to assess the connotation between measured variables. *P*-value ≤ 0.05 is considered significant.

## Results

**Fig. 1:**
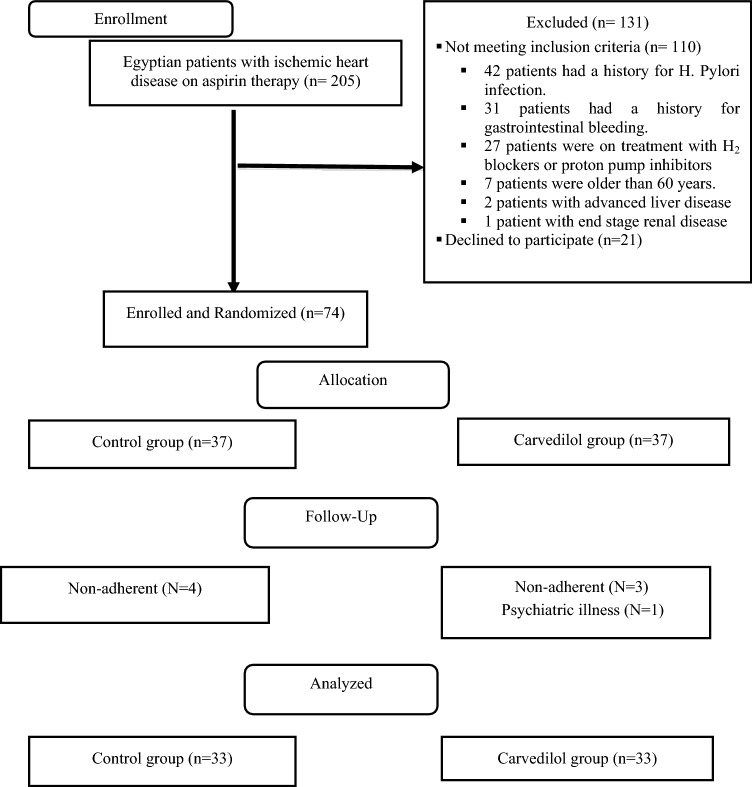
Participants flow-chart

Out of 205 patients with IHD on aspirin therapy screened for eligibility, 131 patients were excluded, 110 did not meet the inclusion criteria, while 21 refused participation. Consequently, only 74 participants were selected, randomized to 1:1 ratio, and allocated into two groups (37 per group). Throughout the follow-up period, 7 patients were non adherent (4 in the control group and 3 in the carvedilol group) and one patient in the carvedilol group was diagnosed with psychiatric illness and then they were excluded from the study. Hence, the final study cohort comprised 66 patients (33 per group) as postulated in Fig. 1.

### Baseline demographic, anthropometric and clinical data

Regarding demographic data and baseline characteristics, no statistically significant differences were noted between both groups in terms of age, gender, weight, height, BMI, smoking habits, and the consumption of dual antiplatelet therapy as shown in Table 1.

**Table 1:** Patients’ demographics and baseline characteristics

Parameter	Control group (*n* = 33)	Carvedilol group (*n* = 33)	*P*-value
Age (years)	57.8 ± 7.1	57.9 ± 5.4	0.948
*Sex*			
Male	23 (69.7%)	22 (66.7%)	0.792
Female	10 (30.3%)	11 (33.3%)	
Height (cm)	162.7 ± 4.9	160.8 ± 5.9	0.157
Weight (kg)	75.5 ± 12.2	75 ± 11.8	0.866
BMI (kg/m^2^)	29 ± 4.5	29 ± 3.8	1
*Smoke*			
Yes	15 (45.45%)	13 (39.39%)	0.621
No	18 (54.54%)	20 (60.6%)	
*Dual antiplatelet*			
Yes	13 (39.39%)	10 (30.3%)	0.441
No	20 (60.6%)	23 (69.7%)	

Numerical data are presented as mean ± standard deviation; nominal data are presented as number (percent)

cm: centimeter; kg: kilogram; BMI: body mass index; m^2^: meter square

*p* ≤ 0.05 is statistically significant

### Effect of study medications on the biomarker serum levels

At baseline, the statistical analysis using unpaired t-test revealed absence of significant variations between both groups (*P*_2_ > 0.05) regarding the serum level of the assessed biological markers as demonstrated in Table 2.

**Table 2:** The serum levels of the assessed biological markers for the two study groups

Parameter	Control group (*n* = 33)	Carvedilol group (*n* = 33)	*P*_2_-value
MDA (nmol/mL)			
Baseline	1.1 ± 1.09	1.57 ± 1.78	0.20
After treatment	3 ± 2.43	1.42 ± 1.65	0.003*
*P*_1_-value	< 0.001*	0.723	
PGE2 (pg/ml)			
Baseline	611 ± 110	619 ± 75.3	0.731
After treatment	552.26 ± 115.5	642.2 ± 94	< 0.001*
*P*_1_-value	0.038*	0.272	
4-HNE (pg/ml)			
Baseline	143 ± 22	142.1 ± 25.5	0.878
After treatment	149 ± 21.7	126.8 ± 23	< 0.001*
*P*_1_-value	0.268	0.012*	
GAS-17 (pg/ml)			
Baseline	302.3 ± 73.2	314 ± 74.3	0.52
After treatment	285.7 ± 58	254 ± 44.3	0.015*
*P*_1_-value	0.311	< 0.001*	

Numerical data was presented as mean ± standard deviation

MDA: malondialdehyde; PGE2: prostaglandin E2; 4-HNE: 4-hydroxynonenal; GAS-17: gastrin-17

nmol/mL:nanomoles per liter**,** pg/ml: picograms per milliliter

*P* ≤ 0.05 is statistically significant

*P*_1_-value: probability of significance within the same group (before versus after intervention)

*P*_2_-value: probability of significance between the two study groups

*Statistically significant difference

Compared to the baseline data, the study findings demonstrated that, after three months of intervention, the control group exhibited significantly increased serum level of MDA (1.1 ± 1.09 nmol/mL versus 3 ± 2.43 nmol/mL; *P*_1_ < 0.001) and decreased serum level of PGE2 (611 ± 110 pg/ml versus 552.26 ± 115.5 pg/ml; *P*_1=_ 0.038). However, serum levels of 4-hydroxynonenal (4-HNE) (143 ± 22 pg/mL vs. 149 ± 21.7 pg/mL; *p* = 0.268) and gastrin-17 (GAS-17) (302.3 ± 73.2 pg/mL vs. 285.7 ± 58 pg/mL; *p* = 0.311) showed no statistically significant alterations. Regarding carvedilol group, our findings indicated significant decrease in the of 4-HNE serum levels (142.1 ± 25.5 pg/ml versus 126.8 ± 23 pg/ml; *P*_1_ = 0.012) and the GAS-17 serum levels (314 ± 74.3 versus pg/ml 254 ± 44.3 pg/ml; *P*_1_ < 0.001) compared to the baseline data. However, no significant variations in the serum levels of both MDA (1.57 ± 1.78 nmol/mL versus 1.42 ± 1.65 nmol/mL; *P*_1_ = 0.723) and PGE2 (619 ± 75.3 pg/ml versus 642.2 ± 94 pg/ml; *P*1 = 0.272) was reported, as shown in Table 2.

The post-intervention comparison between the two groups showed that, in contrast to the control group, the carvidelol group exhibited a significant reduction in the serum levels of MDA (3 ± 2.43 nmol/mL versus 1.42 ± 1.65 nmol/mL; *P*_2_ = 0.003), 4-HNE(pg/ml) (149 ± 21.7 versus 126.8 ± 23; *P*_2_ < 0.001) and GAS-17 (285.7 ± 58 pg/ml versus 254 ± 44.3 pg/ml; *P*_1_ = 0.015). This was associated with a significant rise in the PGE2 serum levels (552.26 ± 115.5 pg/ml versus 642.2 ± 94 pg/ml; *P*_2_ < 0.001) as illustrated in Table 2.

### Clinical assessment

At baseline, the statistical analysis revealed absence of significant variations between both groups (*P*_2_ > 0.05) concerning the SAQ-7 score (63.3 ± 20.6 versus 63 ± 25; *P*_2_ = 0.957) and SAGIS questionnaire score (5 ± 3.1 versus 6.03 ± 3.1; *P*_2_ = 0.18) as demonstrated in Table 3. The study results demonstrated that after three months of intervention, the control group showed non-significant variation in both SAQ-7 score (63.3 ± 20.6 versus 62.5 ± 22; *P*_1_ = 0.879) and SAGIS questionnaire score (5 ± 3.1 vs 6.3 ± 6.1; *P*_1=_0.279). Conversely, carvedilol group showed significant increase in SAQ-7 score (63 ± 25 vs 75.43 ± 26; *P*_1_ = 0.039) and significant decrease in SAGIS questionnaire score 6.03 ± 3.1 versus 4 ± 4.2; *P*_1_ = 0.029) after three months of intervention, as shown in Table 3.

**Table 3:** Clinical parameters for the two study groups

Parameter	Control group (*n* = 33)	Carvedilol group (*n* = 33)	*P*_2_-value
*SAQ-7*			
Baseline	63.3 ± 20.6	63 ± 25	0.957
After treatment	62.5 ± 22	75.43 ± 26	0.033*
*P*_1_-value	0.879	0.039*	
*SAGIS*			
Baseline	5 ± 3.1	6.03 ± 3.1	0.18
After treatment	6.3 ± 6.1	4 ± 2.4	0.04*
*P*_1_-value	0.279	0.029*	

Numerical data was presented as mean ± standard deviation

SAQ-7: seattle angina questionnaire; SAGIS: structured assessment of gastrointestinal symptoms.

*P* ≤ 0.05 is statistically significant

*P*_1_-value: probability of significance within the same group (before versus after intervention)

*P*_2_-value: probability of significance between the two study groups.

*Statistically significant difference

The comparison between the two group after intervention revealed that, carvidelol group showed a significant increase in in SAQ-7 score (62.5 ± 22 vs. 75.43 ± 26; *P*_2_ = 0.033) and a significant decrease in SAGIS questionnaire score (6.3 ± 6.1 vs. 4 ± 4.2; *P*_2_ = 0.04) compared to the control group, as illustrated in Table 3.

### Drug safety and tolerability

During the study duration, patients were monitored via weekly telephone calls and scheduled hospital visits to judge treatment adherence and document any potential medication-linked adverse effects. The safety monitoring revealed no serious drug-related complications among study participants. Most adverse effects reported were mild and controllable. The statistical analysis revealed absence of significant variation in the reported adverse effects between the two groups which included dizziness (6.1% vs 3%; *P* = 0.55) and headache (9.1% vs 0%; *P* = 0.08). However and compared to the control group, the carvedilol group exhibited significantly lower incidence of cough (12.1% vs 0%; *P* = 0.041) and GIT upset (30.3% vs 9.1%; *P* = 0.032) as shown in Table 4.

**Table 4:** The reported adverse effects for the two study groups

Adverse effects	Control group (*n* = 33)	Carvedilol group (*n* = 33)	*P*_2_-value
Cough			
Yes	4 (12.1%)	0 (0%)	0.041*
No	29 (87.9%)	33 (100%)	
Dizziness			
Yes	2 (6.1%)	1 (3%)	0.55
No	31 (93.9%)	32 (97%)	
Headache			
Yes	3 (9.1%)	0 (0%)	0.08
No	30 (87.9%)	33 (100%)	
GIT upset			
Yes	10 (30.3%)	3 (9.1%)	0.032*
No	23 (69.7%)	30 (90.9%)	

Data are expressed as number and percentage

GIT: gastrointestinal tract;

*P* ≤ 0.05 is statistically significant

*Statistically significant difference

## Discussion

This study evaluated the gastroprotective efficacy of carvedilol in individuals with ischemic heart disease (IHD) receiving aspirin treatment. The comparable baseline characteristics between both study groups and any post-intervention differences observed can be reliably attributed to the pharmacological effects of the study medications rather than to interindividual variability.

Following a three-month intervention, the carvedilol group demonstrated statistically significant decreases in serum concentrations of MDA and 4-HNE relative to the control group. This supports the notion that carvedilol may possess antioxidant properties (Toyoda et al. 2020) and suggests a link between oxidative stress and aspirin-induced gastrointestinal damage (Verma and Kumar 2019). It has been demonstrated that the α1/β adrenoceptor antagonist carvedilol lowers blood pressure and shear stress on vessel walls, which in turn inhibits the renin-angiotensin system, NADPH oxidase, and superoxide generation (Toyoda et al. 2020). Carvedilol thus offers both direct and indirect antioxidant effects (Osman et al. 2017; Ahmed et al. 2020).

The current investigation revealed that patients in the control group experienced a statistically significant reduction in PGE2 levels compared to baseline data. However, patients receiving carvedilol therapy showed significant elevation in PGE2 compared to the control group. This finding underscores the preventive function of carvedilol on the gastric mucosa and highlights the detrimental effects of aspirin on gastric mucosa (Wehland et al. 2012). The reduced PGE2 levels result in decreased gastric mucus, HCO₃⁻ secretion, and gastrointestinal blood flow, ultimately leading to gastrointestinal injury (Wang et al. 2021).

Carvedilol has been reported to have gastroprotective effects, mediated primarily through its antioxidative and anti-inflammatory properties (Ahmed et al. 2020), which modulate the nuclear factor kappa-B (NF-κB)/cyclooxygenase-2 (COX-2)/inducible nitric oxide synthase (iNOS) pathways (Dandona et al. 2007). These previously reported findings could justify our result, which revealed that carvedilol therapy is associated with a statistically significant decrease in GAS-17 as compared to the control group after the treatment period. The reduction in gastrin production and its impact on the stomach lowers the risk of peptic ulcers and aids in the healing of those who already have ulcers (Guthrie 2011). After the treatment period, the carvedilol-treated group showed a significantly higher SAQ-7 score as compared to the control group. Moreover, the carvedilol-receiving group revealed a significant decline in the structured assessment of gastrointestinal symptoms (SAGIS) questionnaire score. These aforementioned findings indicate the ability of carvedilol to enhance quality of life and reduce the severity of gastric symptoms in patients with IHD on aspirin therapy. The beneficial effects of carvedilol on the SAGIS questionnaire are attributed to its gastroprotective effects, which are maintained through its favorable effects on MDA, 4-HNE, PGE2, and GAS-17. Our finding regarding the effect of carvedilol on both the SAQ-7 score and the SAGIS questionnaire aligns with previous reports (Oh et al. 2016). This study demonstrated that carvedilol has a favorable tolerability and safety profile. No significant adverse reactions were observed in either of the two study groups. The predominant adverse effects recorded were mild and manageable. No significant variance was observed between the two groups regarding the reported adverse effects, with the exception of cough and gastrointestinal upset. The carvedilol group exhibited a markedly reduced incidence of cough and gastrointestinal upset relative to the control group. This finding corroborates the safety and tolerability of carvedilol, consistent with earlier studies (Krum 2004).

This study has several significant strengths, including its randomized controlled parallel design and its status as the first clinical investigation assessing the efficacy and safety of carvedilol as a cardioprotective agent in patients with ischemic heart disease undergoing aspirin therapy. However, the study presents certain limitations, such as a limited sample size and an open-label design. Consequently, additional extensive studies are still required.

## Conclusion

In this randomized controlled parallel study, the carvedilol-treated group showed significantly lower levels of malondialdehyde (MDA), 4-hydroxynonenal (4-HNE), and gastrin-17 (GAS-17), which was associated with a significantly higher level of prostaglandin E2 (PGE2). These favorable impacts on the biological markers involved in the pathogenesis of gastric injury were associated with clinical improvement, as indicated by the structured assessment of gastrointestinal symptoms (SAGIS) questionnaire and the Seattle Angina Questionnaire (SAQ-7) score. These overall findings suggest that carvedilol may exert significant clinical benefits as a gastroprotective agent in IHD patients receiving aspirin treatment, in addition to its favorable effects on ischemic heart disease. However, future large-scale and more longitudinal studies are still necessary to validate our promising results.

## Data Availability

Data are available upon reasonable request from the corresponding author.
